# Learning disability nurse provision in children’s hospitals: hospital staff perceptions of whether it makes a difference

**DOI:** 10.1186/s12887-019-1547-y

**Published:** 2019-06-11

**Authors:** Kate Oulton, Jo Wray, Angela Hassiotis, Charlotte Kenten, Jessica Russell, Irene Tuffrey-Wijne, Mark Whiting, Faith Gibson

**Affiliations:** 1 Centre for Outcomes and Experience Research in Children’s Health, Illness and Disability (ORCHID), Great Ormond Street Hospital for Children NHS Foundation Trust, Level 4, Barclay House, 37 Queen Square, London, WC1N 3BH England; 2 UCL Division of Psychiatry, London, 6th Floor, Maple House, 149 Tottenham Court Road, London, W1T 7NF England; 3 Faculty of Health, Social Care & Education, Cranmer Terrace, Kingston University & St George’s, University of London, 6th floor Hunter Wing, London, SW17 0RE UK; 4 Health Research Building, University of Hertfordshire, College Lane Campus, Hatfield, Hertfordshire, AL10 9AB England; 5 School of Health Sciences, Faculty of Health and Medical Sciences, University of Surrey, Guildford, Surrey GU2 7XH England

**Keywords:** Learning disability nurse provision, Intellectual disability, Workforce planning, Mixed methods, Health services research

## Abstract

**Background:**

In response to multiple United Kingdom investigations and inquiries into the care of adults with learning disabilities, Mencap produced the Getting it Right Charter which campaigned for the appointment of a Learning Disability Liaison Nurse in every hospital. More recent best practice guidelines from the Care Quality Commission included the need for all children’s units to have access to a senior learning disability nurse who can support staff and help them manage difficult situations. However, little evidence exists of the extent of learning disability nurse provision in children’s hospitals or the nature and impact of this role. Here we report selected findings from a national mixed methods study of hospital care for children and young people with and without learning disabilities in England. The extent of learning disability nurse provision in children’s hospitals is described and perceptions of staff working in hospitals with and without such provision is compared.

**Methods:**

Semi-structured interviews were conducted with senior staff across 15 children’s hospitals and an anonymous survey was sent to clinical and non-clinical staff with patient (children and young people) contact within these hospitals. The survey focused on six different elements of care for those with and without learning disability, with additional questions concerning identifying and tracking those with learning disabilities and two open-ended questions.

**Results:**

Forty-eight senior staff took part in interviews, which included a subset of nine nurses and one allied health professional employed in a dedicted learning disability nurse role, or similar.

Surveys were completed by 1681, of whom 752 worked in a hospital with dedicated learning disability nurse provision. We found evidence of limited and varied learning disability nurse provision which was valued by hospital staff and shown to positively impact their perceptions of being capable to care for children and young people with learning disabilities, but not shown to increase staff perceptions of capacity or confidence, or how children and young people are valued within the hospital, their safety or access to appointments.

**Conclusion:**

Further consideration must be given to how learning disability nurse roles within children’s hospitals are best operationalised in practice to have the greatest impact on staff and families, as well as how we monitor and evaluate them to ensure they are being utilised effectively and efficiently.

**Trial registration:**

The study has been registered on the NIHR CRN portfolio 20,461 (Phase 1), 31,336 (Phases 2–4).

## Background

In 2007, MENCAP published ‘Death by Indifference’ [1] a Report focused upon the deaths of six people with learning disability (internationally referred to as Intellectual Disability (ID)) in hospital, which highlighted inequalities in health care and laid a charge of institutional discrimination against the National Health Service (NHS). Three years later, 200 NHS Trusts and Organisations signed the MENCAP ‘Getting it Right Charter’ [2], which campaigned for the appointment of a Learning Disability Liaison Nurse (LDLN) in every hospital. Whilst subsequent inquiries and recommendations about the care of people with learning disability in hospital has focused on adults, rather than on the specific needs of children and young people, there is evidence that this population routinely experience particularly poor health outcomes. For example, a review of the evidence on the prevalence and determinants of health conditions and impairments among children and young people with learning disability in the United Kingdom (UK) [3], revealed that the risk of children being assessed to have fair/poor general health by their main carer was 2.5–4.5 times greater for those with learning disability compared to those without [4, 5]. Children and young people with learning disability are also almost twice as likely to report three or more health problems and more than four times as likely to be diagnosed as having a psychiatric disorder than children without a learning disability [4, 6] It is also recognised that such conditions can remain unidentified or misattributed to the person’s learning disabilites, a process known as diagnostic overshadowing [7]. More recently, best practice guidelines issued by the Care Quality Commission (CQC) [8] have brought the needs of children and young people with learning disability to the fore, by calling for *“all children’s units to have access to a senior learning disability nurse who can provide information, advice and support to health care staff involved in the care of such children and who can help manage difficult situations”* (p65). As Glasper [8] reports, this came following a series of CQC inspections highlighting their concern about the *“plight”* of this group of patients (p63).

Little evidence exists of the extent of learning disability nurse provision in children’s hospitals or the nature and impact of this role. A recent NHS benchmarking exercise [9] aimed at providing a *“broad assessment of the state of NHS learning disability services” (*p3) revealed important information about inpatient and community adult provision and community children’s provision. However, no data concerning children’s inpatient learning disability service provision was provided. As highlighted in the Royal College of Nursing [10] position statement on the role of the learning disability nurse, *“National work needs to be undertaken by each UK country as a matter of priority to profile the existing learning disability nursing workforce and identify future requirements*” (p9). A Department of Health commissioned review by the National Council for Disabled Children [11] revealed a number of staffing issues related to the care of children and young people with complex needs and behaviour that challenges involving mental health problems and learning disabilities and/or autism. A key finding was the lack of recognition and value placed upon the specific skills needed for working with these children, with no professional group identifying themselves as being wholly trained in one or more of their needs. Furthermore, specific issues surrounding the recruitment of nurses with learning disability education and training were identified including the possibility that “*it was only when they were on shift that care plans for this group were implemented*” (p28). A need to understand the staff skill gaps in respect of caring for these children and take necessary action was highlighted.

Brown et al. [12] conducted a mixed methods impact and outcome study of adult LDLN services in south-east Scotland and developed a conceptual model comprising seven elements of the LDLN role: advocating, collaborating, communicating, educating, facilitating, influencing and mediating and three dimensions of influence: clinical, educational and strategic. All stakeholders reported highly valuing the LDLN services. Liaison nurses were seen as playing a fundemental role in raising the profile and status of people with learning disability and through their expert skills and knowledge contributing to the effectiveness of systems and process and achieving person-centred outcomes. Brown and colleagues [13] go on to discuss the role of LDLN in identifying and making reasonable and achievable adjustments within the general hospital setting, and describe them as being *“one of the solutions to achieving safe, effective and person-centred care”* (p1560). What we do not know is whether the model of LDLN developed by Brown et al. [12] is applicable for use in children’s hospitals in England.

Tuffrey-Wijne et al. [14] conducted a mixed-methods study of six acute hospitals in England, in order to identify the factors that promote a safer hospital environment for adults with learning disabilities. Three of the participating hospitals employed a hospital-based LDLN; two worked closely with community-based LDLNs; one did not have an LDLN. The researchers found that LDLNs were the main enablers of safe and good-quality healthcare for people with learning disabilities in hospitals. The LDLN was pivotal in identifying patients with learning disabilities within the hospital; identifying individual needs for reasonable adjustments; and ensuring implementation of reasonable adjustments. The effectiveness of LDLN nurses was found to be dependent on strong support for the role at senior management level; authority within the LDLN role to make decisions that change patient pathways; and high visibility and availability within the hospital, including sufficient cover for absence. Without these, there was a risk of the role being marginalised. A recent audit of the quality of inpatient care for adults with intellectual disability in the UK [15] similarly found that the presence of a LDLN contributed to improved care for these patients, including an increased likelihood of them having an epilepsy risk assessment and greater use of a hospital passport. However, the study was underpowered to draw definitive conclusions about the impact of LDLNs, with the need for further work to confirm the benefits of the role being identified.

The current manuscript relates to a national 4-phase mixed methods study of hospital care for children and young people with and without learning disabilities receiving care in twenty-four hospitals in England [16]. By children with learning disabilities we are referring to those with reduced intellectual functioning resulting in *“diminished ability to adapt to the daily demands of the normal social environment”*[17]. This does not include children with learning difficulties that may impair educational attainment, e.g. processing problems, but who are within the average range of intelligence or those with developmental delay who are late in reaching some or all of their developmental milestones.

### Aim

Phase 1 of this study sought to understand the organisational context for healthcare delivery to children and young people with learning disabilites, and compare staff views of their ability to identify and meet the needs of both those with and without learning disabilities [18]. Two objectives from this phase of work form the basis of this paper:

1. To identify the nature and extent of dedicated learning disability nurse provision in children’s hospitals,
2. To compare perceptions of staff working in hospitals with dedicated learning disability nurse provision with those working in hospitals without.

Data related specifically to the ‘flagging’ of children and young people with learning disabilities and the processes and practices of involving and engaging with them are reported separately.

### Hypotheses

1. Staff who work in hospitals with a dedicated learning disability nurse will have greater capability and confidence to meet the needs of children and young people with learning disability than staff working in those without a dedicated learning disability nurse.
2. Staff who work in hospitals with a dedicated learning disability nurse are more likely to perceive their hospital as valuing children and young people with learning disability than staff working in hospitals without a dedicated learning disability nurse.

### Sample and setting

Phase 1 of this study was conducted in 15 children’s hospitals in England and nine non-children’s hospitals. This paper reports on the data collected from children’s hospitals only. The local Principal Investigator at each hospital site was asked to identify at least two senior staff well placed to answer questions about the organisational context for healthcare delivery to children and young people with learning disabilites.

## Methods

Semi-structured interviews were conducted with senior staff either in person (*n* = 3) or over the telephone (*n* = 45) across the 15 children’s hospitals. With permission, interviews were recorded and transcribed verbatim. As part of the interviews, participants were asked about any dedicated learning disability nurses in their hospital, including their job title, hours and remit. Further clarification was sought from the local Principal Investigator at each hospital if the information provided was unclear or inconsistent.

An anonymous survey was also sent to clinical and non-clinical hospital staff working with children and young people. The survey focused on six different elements of care (capability, capacity, confidence, safety, values, and access) for those with and without learning disability, with additional questions regarding processes used for identifying and tracking those with learning disability (see Table 1). Likert scales were used for each question, with the majority of questions rated on a 5-point scale of ‘strongly agree’ to ‘strongly disagree’. Two open-ended questions were included to understand staff perspectives about what their hospital does well to support children and young people with and without learning disability and what could be done better. A fuller description of the methods has been reported elsewhere, including a copy of the interview schedule and full survey [17].

**Table 1:** Staff survey questions related to children and young people with learning disabilities grouped by domains

Domain	Question/statement	α
Capability	1. I have the necessary knowledge and skills to meet their needs	.843
	2. I have the necessary training to meet their needs	
	3. I feel able to identify what reasonable adjustments are needed	
Capacity	4. I routinely have access to necessary resources to meet their needs	.807
	5. I routinely have access to additional specialist support to meet their needs	
	6. I routinely have access to additional learning disability (LD) specialist staff to meet their needs	
	7. I work in an environment that is designed to take into account their individual needs	
	8. I feel confident that any reasonable adjustments will be accommodated in a timely way	
Confidence	9. How confident are you about identifying that a child/young person (CYP) in your care/who you meet has a learning disability?	.753
	10. I feel confident to communicate effectively with them	
	11. I feel confident to assess and manage pain	
	12. I feel confident to safely manage challenging behaviour	
Safety	13. I work in an environment that is safe for meeting their needs	.784
	14. I am always able to deliver safe care	
Values	15. I feel CYP with LD are always treated with dignity and respect	.798
	16. Overall, I think my Trust values CYP with LD	
Access	17. In my hospital, CYP with LD have appropriate access to: • Medical care and equipment • Educational provision • Play and stimulation • Appointments (including double, first/last, flexible appointments)	

### Data analysis

Staff interviews and free text responses were analysed thematically using NVivo 11 and the Framework approach [18–20]. Data relating specifically to aspects of the learning disability nurses’ role were collated and mapped onto an existing framework for defining the role of the learning disability nurse developed by Brown et al. [12]

Descriptive statistics were used to characterise the sample. Composite variables were computed to represent capability, capacity, confidence, safety, values and access to appointments, as outlined in Table 1. All composite variables had acceptable internal reliability with Cronbach alpha values > 0.7. Items about access to medical care, education and play were analysed individually. Responses from participants from hospitals with learning disability nurse provision were compared with responses from participants from hospitals without learning disability nurse provision for all six domains and for the appointments and individual items in the access domain. Having assessed the normality of the data, either Mann-Whitney or t-tests for two independent samples were used. A Bonferroni correction for multiple comparisons was applied and a *p* value of .005 was considered significant for all analyses. All data were analysed using SPSS version 22.

## Results

The sample of 48 senior staff who took part in interviews included a subset of nine nurses and one allied health professional employed in a dedicted learning disability nurse role, or similar. Surveys were completed by 1681 staff, 752 of whom worked in a hospital with dedicated learning disability nurse provision (Table 2).

**Table 2:** Staff survey respondents across 15 children’s hospitals in England

	Number of participants	Doctor	Nurse	AHP	Ancillary staff	Job title Missing
Hospitals with dedicated learning disability nurse provision	752	122	357	146	120	7
Hospitals without dedicated learning disability nurse provision	929	154	484	115	168	8
Total	1681	276	841	261	288	15

*AHP Allied Health Professional.*

For clarity when reporting, the *extent* of learning disability nurse provision in children’s hospitals in England will be presented first, followed by qualitative findings about the *nature* of such provsion (objective 1), taken from the subset of ten interviews and the complete set of free-text survey responses. Finally, the quantitative findings from the survey, comparing perceptions of staff working in hospitals with/without dedicated learning disability nurse provision, are reported (objective 2).

### Extent of learning disability nurse provision

Learning disability nurse provision was in place in eight (53%) children’s hospitals. As shown in Table 3, provision varied across sites in terms of numbers of staff, tenure and remit. Furthermore, job titles varied considerably including, for example, senior nurse, specialist nurse, lead nurse and liaison nurse. Two of the thirteen nurses identified were not learning disability trained, but had a specific remit for the care of children with learning disabilites.

**Table 3:** Learning disability nurse provision in children’s hospitals in England

Site	Number of LD Nurses	Hours	Coverage	Remit
1	One	Part time	Hospital	Children
2	Two^a^	Part time	Hospital	Children
3	Two	Two full time	Hospital and Community	Children and Adults
4	One	Part time	Hospital	Children and Transition
5	One	Full time	CAMHS^b^	Children
6	Three^a^	Two full time One unknown	Hospital and Community	Children
7	Two	Two full time	Hospital	Children
8	One	Full time	Hospital	Children and Adults

^a^Includes non-learning disability trained nurses with a remit for learning disability care

^b^Child and Adolesclent Mental Health Service

### Nature of learning disability nurse provision

Phase 1 data collection did not include a formal evaluation of the role of the learning disability nurses in children’s hospitals. However, interviews with a subset of nine nurses and one allied health professional employed either in a dedicted learning disability nurse role (*n* = 8), or similar (*n* = 2), revealed elements of how the role is operationalised in practice as well as valuable insights about workforce and organisational culture. These participants are referred to hereafter as ‘nurses’ to prevent identification. The hospitals where they worked were at various stages of development in terms of their organisational and practical approach to the care of children and young people with learning disabilities including those drawing on well established support for children with autism, or adapting and applying practices for adults with learning disabilities, to those without these foundations to build upon.

#### Workforce

A key finding was the varying breadth and depth of provision across children’s hospitals, with some nurses working in particular clinical areas such as neurodisability or CAMHS, whilst others had Trust wide responsibility, and some working with particular groups of children e.g. those with autism (including those with learning disabilities) or those who were transitioning to adult services. The degree to which nurses understood what was in place at the organisational level invariably differed with some being knowledgeable only about their specific area of practice. For example, one nurse who was asked about how children with learning disabilities are identified within her Trust said, *“I can’t speak for the Trust. I don’t know anything about outside of this service in the Trust”.* It was also apparent that the way children are defined in relation to the nature of their ‘disability’ could have implications for how some learning disability nurses worked:

*“We have a learning disability policy for adult patients that includes children but I don’t specficially work with it because I have a remit of children with disabilities which includes physical disabilities as well”.*

This nurse went on to explain that children with learning disabilities who transition to adult services will get ‘picked up’ by the adult learning disability team but *“they only cover learning disability and not their physical needs”,* which has implications for ward staff who *“are expected to manage those independently”*.

The lack of learning disability nurses employed at a senior level was criticised by one nurse who described it as, *“a typical indictment that people with a learning disability* … *and the* [lack of] *value of their lives”*. This staff member went on to highlight that learning disability nurses across the country working at a more junior level cannot make the same impact, and *“they’re not able to think fiscally about how they’re doing”.*

Many nurses talked about the value of having learning disability champions or link leads within the Trusts, although it was reported by one participant that many in their organisation were not actively engaging with the role.

#### Culture

A number of nurses talked about the culture of their organisation regarding the care of children and young people with learning disabilities. There was widespread recognition that there was a lot of work to do and that things were far from perfect. However, some sensed a growing willingness and commitment from within their organisation to get it right for this population, with the belief that, *“if we get it right for children with learning disabilities or additional needs, we’ll get it right for everybody hopefully”.* This nurse went on to describe the open and honest culture where s(he) worked, built around listening to others and the *“massive swell of people wanting to do better … get involved… and be upskilled”* to improve care for patients. This drive for improvement was also seen to be important in terms of families’ emotional well-being:

*“We have very good support from people quite high up … we need to be doing more, we need to be reasonably adjusting. We want to listen, we want to improve things. We want children, their parents or carers to want to come to this Trust … not be frightened to come back”.*

Whilst some nurses described their organisation as being at an embryonic stage in terms of their thinking around the care of children with learning disabilities, others talked about positive practices becoming more embedded over time:

*“You do hear stories now of people, nurses, doing those adjustments themselves. It doesn’t always have to come from us. It’s not perfect … but I think the awareness in the hospital is certainly a lot more than when [name of colleague] started five or six years ago”.*

One nurse held negative views about their organisation and their approach to the care of children with learning disabilities. They described them as having a long-way to go in this area, in contrast to how medical care was being delivered which was seen as cutting edge. The perception was that the organisation was risk averse and that involvement and engagement with children with learning disabilities was tokenistic, stating that, *“there are an awful lot of bigots in disguise within the hospital … the sugar friendly people who will say to you, there’s no point having an alert system”.* This nurse did go on to say that there had been a change of senior staff within the Trust, and that barriers were subsequently *“falling away”.*

### Role descriptions

**Fig. 1:**
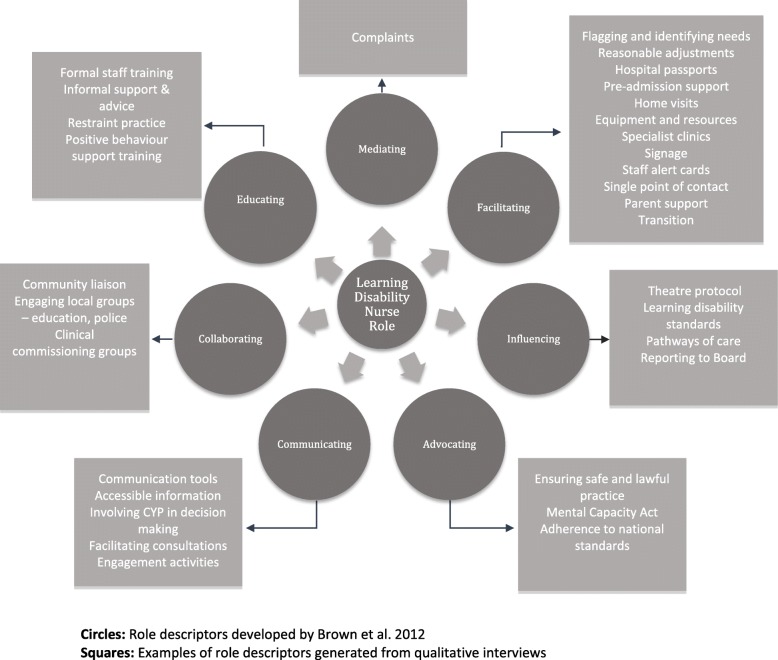
Learning disability nurse role descriptors

Interviews with nurses revealed a clear overlap between the nature of their role and the role descriptors provided by Brown et al. [12] (see Fig. 1).

Their role as facilitators was apparent through their direct contact with families or supporting staff, especially in relation to supporting reasonable adjustments. The need for individualised care for these patients was described, for example in relation to hospital appointments, the physical and sensory environment, waiting and safety. A key issue described was the challenge in acquiring the necessary information to plan and make reasonable adjustments in advance rather than adopting a reactive approach at the *“point of contact”.* As one nurse said,

*“If a family can give us plenty of notice once they get their letter then we can start making adjustments but what we are not good at is picking up from the moment … to be proactive and to say ‘Hi, what do you need us to do?’, because children are varied and they change so quickly, so we tend to rely on families getting in touch with that”.*

This nurse went on to highlight the challenge of ‘one off’ or emergency admissions because parents will not be driven to share information about their child in the same way as those with previous negative experiences to draw on:

*“Most children with disabilities when coming in normally had something missing or went wrong before which they want to make sure doesn’t happen again”*.

The impact that negative experiences can have on future decisions is highlighted particularly clearly by another nurse in the following quote:

*“Lots of parents are reluctant to come into our emergency department … we do get issues from that, which means that some children, sometimes aren’t presenting or they’re quite ill, but for some reason, doing what they can at home to avoid hospital admission”*.

Their role in educating staff and students, both in providing formal and informal training, was described. When asked about meeting the needs of children with learning disabilities, one nurse said:

*“We’ve still not got it right by all means because we’re massive and the rootcause really is education and people learning and listening to families and I can hear our families saying, ‘if we get it wrong, we get it wrong big’”.*

This nurse went on to highlight the importance of staff not only receiving *core* training, but then being able to apply it in practice, for example, in the case of knowing what communication aids to use and where to find them for a non-verbal patient brought in as an emergency on a Saturday night.

The link between training and confidence was highlighted, as well as the need to empower and upskill professionals. At one hospital, staff across the accident and emergency department, reception areas and volunteers were, *“being trained in things like positive behaviour support, trying to make sure that people are acting proactively and helping the child to manage their own behaviour in relation to what’s going on in the environment”*.

The decision in one particular hospital to encompass aspects of learning disabilities into existing policies, rather than have a standalone policy, was to encourage staff to be less reliant on the learning disability nurses. In terms of communication, nurses talked about having accessible information, although hospitals varied in terms of what they used, particularly in relation to knowledge and use of easy read materials. The use of symbols and photographs was frequently reported, including making photographic journeys of the hospital available to patients in advance of their admission, creating visual timetables and improving signage.

Particular issues were described in relation to the use of health/hospital passports. As one nurse said, *“we get complaints from parents that even when a passport has been pushed through to somebody’s hand, that they feel it hasn’t been read”* . This nurse goes on to explain that families often do not come in with passports, they do not have time to access and complete them and there are barriers for parents for whom English is not their first language. There are also issues with staff not finding the time or being able to access and print off hospital passports from the website to give to families. A similar issue was raised by another nurse who said:

*“We’ve rolled our the health passport but that’s in relation to the individual practitioner … it links to staff knowledge, time and accessibility of the docment and the willingness of the practioner to read or take information from it … trying to make sure that’s standard practice at the moment”.*

Collaborating with community based professionals appeared to be a significant part of these nurses’ role, particularly with those working in special needs schools. Advocacy and mediation were only touched upon by a couple of nurses, one who highlighted the role parents played in advocating for their child and “*fighting to make sure their child is seen”* and another who talked about her role in managing complaints. Some of those who were interviewed spoke about the strategic element of their role, and trying to influence the culture and practices at the organisational level, through the creation of systems, pathways and policies.

### Survey comments

Data extracted from the open survey questions about what staff thought their hospital did well to support the care of children and young people with learning disabilities (*n* = 562 responses) and what could be done better (*n* = 597 responses), revealed the value many placed on having access to someone with expertise and experience in learning disability. In terms of what their hospital did well, 65 comments were made specifically about the provision of learning disability nurse(s) who were described as supporting families, influencing the corporate agenda, providing training and support for staff, flagging and alerting staff about patients, developing external collaborations, using hospital passports, advocacy, and facilitating in terms of transition to adult services and access to services. A further 16 comments highlighted the importance of having learning disability link nurses or ‘champions’ on the ward, for example, *“We have link nurses in all departments so there is always someone to refer to and we meet regularly to discuss what we are doing and ideas for what we could be doing”.*

In terms of what their hospital could do better to support children and young people with learning disabilities and their families, the majority of comments were related to staff having the appropriate experience (*n* = 48) and training (*n* = 127) to meet the needs of this population, for example, *“having greater number of Makaton trained staff” and “staff with skills to communicate with children with learning disability”.* One respondent commented on the impact that not having sufficiently trained staff could have on the role of parents in hospital:

*Provide more training for staff, especially on learning disabilities and challenging behaviour. I feel we are just left to get on with it, with most of us not having the correct training on how to deal with these patients. Often we ask the parents to stay and if they can’t we often try to provide 1–1 care but this wouldn’t be until the next shift.*

Many comments (*n* = 60) were also made about the need for patients with learning disabilities to be identified, so that staff *“can put things in place before they arrive, rather than doing and learning as the day goes on”*.

### Quantitative findings

The data did not meet assumptions for normality other than for the question about capacity.

Staff working in hospitals with a dedicated learning disability nurse were more likely to have been given information about how to *define* learning disability than staff working in hospitals without a dedicated learning disability nurse (Z = − 2.744, *p* = .006) but this difference was no longer significant after a Bonferroni correction had been applied. Furthermore, those working in hospitals with a dedicated learning disability nurse were no more likely to report being routinely informed that a child/young person has a learning disability than those working in hospitals without a dedicated learning disability nurse.

Our first hypothesis was not supported. Rather we found that staff who worked in hospitals with a dedicated learning disability nurse *did not* report higher levels of confidence [Q9–12] (z = .324, *p* = .746) or capacity [Q4–8] (z = 1.944, *p* = .052) to meet the needs of children and young people with learning disabilities than staff working in hospitals without a dedicated learning disability nurse. The two groups did differ in terms of perceptions of capability (z = 2.156, *p* = .031) but this difference was no longer significant after the Bonferroni correction was applied. Similarly, although the groups did differ in perceptions of capacity to meet the needs of children and young people with learning disabilities (t = 2.054; *p* = .040) the difference was not significant after application of the Bonferroni correction,

With regards to our hypothesis that staff who work in hospitals with a dedicated learning disability nurse are more likely to perceive their hospital as valuing children and young people with learning disability [Q15–16] than staff working in those without a dedicated learning disability nurse, no significant differences were found. A similar pattern was seen with regard safety [Q13–14] (Z = −.730, *p* = .466).

In terms of staff perceptions about the access that children and young people with learning disability have to hospital based education (z = −.673, *p* = .501), medical care (z = − 1.494, *p* = .135), play facilities (z = − 1.633, z = .102) and first/last (z = 1.067, *p* = .286) or flexible (z = 0.718, *p* = .473) appointments, there were no significant differences between those working in hospitals with and without dedicated learning disability nurse provision.

## Discussion

In terms of delivering an equitable service, it is a significant concern that only just over half of children’s hospitals in England have dedicated learning disability nurse provision in place and for those that do, there is considerable variation in terms of the extent and impact. This is despite the Care Quality Commission [8] advocating that all children’s units have access to a senior learning disability nurse. Few sites had more than one or two nurses in place and in some cases their remit was to work across the hospital and the community or cover both child and adult services. With such limited resources and broad scope it is perhaps unsurprising that our hypotheses, about the potential impact of learning disability nurses, were not fully supported. Whilst there was clearly a pattern emerging of learning disability nurses impacting staff at the individual level in terms of their capability [significant difference prior to Bonfferoni correction], they did not appear to have any influence at the organisational level in terms of capacity, safety or values. These quantitative findings link to how some learning disability nurses described their role during interview, in terms of being specific to a particular speciality or patient group, rather than having hospital wide knowledge and responsibilities. As Tuffrey-Wijne [14] found, the effectiveness of LDLN nurses is dependent on authority within the role to make decisions that change patient pathways, as well as high visibility and availability within the hospital, which would indicate that the current provision of learning disability nurses in children’s hospitals is lacking the pre-requisites to be wholly effective. This is particularly concerning in light of our wider Phase 1 findings that hospital staff feel that children and young people with learning disabilities are less safe in hospital and valued less than those without learning disabilities [17]. If it really is only when learning disability nurses are on shift that care plans for these children are implemented [11], then workforce issues need urgent attention.

Qualitative interviews highlighted many positive practices employed by learning disability nurses and others working in a similar role to enhance the care of children with learning disabilities and their families, from facilitating reasonable adjustments at a patient level to creating hospital wide systems, pathways and policies. However, the degree to which individual nurses engaged in different aspects of the role varied. Free text survey comments revealed that staff in children’s hospitals value the support offered by learning disability nurses available to them. However, they also identified a need to be better trained and experienced, highlighting the importance of ongoing education and a fundamental role for learning disability nurses, something they too reocgnised. What also appeared to be key was staff being able to apply what they learnt in practice rather than being reliant on those working in dedicated learning disability roles. Our study did reveal a trend for staff working in hospitals with a dedicated learning disability nurse to be more likely to have been given information about how to define learning disability than staff working in hospitals without a dedicated learning disability nurse [significant difference prior to Bonferroni correction]. However, the former were no more likely to be informed that a child/young person in their care actually has learning disabilities, reflecting a possible disconnect between staff having the necessary knowledge to identify patients with learning disabilities and them using and sharing that knowledge in practice.

We do know that staff find it helpful for children and young people with learning disabilities to be identified and flagged [18], which suggests that the issue is a lack of formal systems and processes for sharing relevant information, and/or a lack confidence in applying knowledge. We did find that, despite the trend for staff working in hospitals with dedicated learning disability nurse provision to feel more *capable* to meet the needs of children with learning disabilities than those working in hospitals without such provision, they did not report feeling more *confident* as might be expected.

### Limitations

The data reported here were collected from 15 children’s hospitals in England and comprised interviews with nine nurses and one AHP working in a dedicated learning disability role (*n* = 8) or similar (*n* = 2) and 1681 responses to the staff survey, including 1159 free-text comments. We did not set out to formally evaluate learning disability nurse provision as this was beyond the scope of our wider project. Hence, interviews were not specifically focussed on the role, but rather knowledge of the systems, practice and policies in place in their organisation. Key questions such as the rationale and decision making behind the delivery of the learning disability nurse service and how much time dedicated learning disability nurses spend undertaking different components of their job description remain unanswered. Furthermore staff interviews were relatively short (30–45 min) due to their clinical commitments. Whilst we ensured that more than one interview was conducted per site to ensure all questions were addressed, this time constraint inevitably placed restrictions on the depth of qualitative data generated.

## Conclusion

This study has contributed to our understanding about the nature and extent of the existing dedicated learning disability nurse workforce in children’s hospitals in England. We have provided evidence of a limited and varied service, valued by hospital staff and shown to positively impact their perceptions of being capable to care for children and young people with learning disabilities. Evidence of a changing culture also emerged and despite recognition that much more needed to be done, there was also a sense of increased willingess and commitment to improve care and outcomes for this population of patients.

However, the provision of learning disability nurses was not shown to increase staff perceptions of capacity or confidence, or how children and young people are valued within the hospital, their safety and access to appointments. In order to understand how we might begin to address these issues and inform workforce planning, the impact of dedicated learning disability nurse provision in specialist children’s hospitals in England requires further investigation. This should include a detailed review of the types of reasonable adjustments required by children and young people with learning disabilities in hospital, the frequency with which these are required and the degree to which they are accommodated. Consideration should also be given to what outcome measures are appropriate to assess effectiveness of the learning disability nurse role, in addition to staff perceptions of capability, confidence and capacity. Measures of patient/family reported experience, safety incidents, complaints and successful patient procedures, for example, may provide a different picture of the impact that learning disability nurses can have.

At present, there is no clear guidance on how many learning disability nurses are needed to deliver safe and effective hospital care to children and young people with learning disabilities or at what level they should be working. This is partly due to the fact that we are lacking evidence of how many children and young people with learning disabilities actually receive hospital care. Hence, further consideration must be given to how these roles are best operationalised in practice to have the greatest impact on families, as well as how we monitor and evaluate them to ensure they are being utilised effectively and efficiently: evidence-based staffing levels are an essential requirement in future workforce planning [21] First, we need to identify staff skill gaps, not only in relation to those with the most complex needs [11], but all patients with learning disabilities, so that efforts and resources can be focused where they are most needed. It will fall to commissioners and service planners to develop and share a clear vision for how they ensure the knowledge and skills of learning disabilities nurses are provided to the right people, in the right places, and at the right time in a way that reflects the values-and rights-based focus of their work [22]. Without this vision and a strong evidence-base on which to make workforce decisions we are in danger of setting up our limited cohort of learning disability nurses who are working in children’s hospitals to fail.

## Data Availability

The datasets generated and/or analysed during the current study are not publicly available as the study is ongoing and further data is still to be reported.

## Abbreviations

CAMHS: Child and Adolescent Mental Health Service
CQC: Care Quality Commission
CYP: Children and Young People
ID: Intellectual Disability
LD: Learning Disability
LDLN: Learning Disability Liaison Nurse
NHS: National Health Service
UK: United Kingdom
